# Individual and unit-level predictors of promotive voice in nursing: a multilevel analysis

**DOI:** 10.1186/s12912-025-03217-3

**Published:** 2025-05-20

**Authors:** Seung Eun Lee, Ja-Kyung Seo, Maura MacPhee

**Affiliations:** 1 College of Nursing, Yonsei University, Seoul, South Korea; 2 Industrial and Organizational Psychology, Yonsei University, Seoul, South Korea; 3 School of Nursing, University of British Columbia, Vancouver, Canada; 4 Mo-Im KIM Nursing Research Institute, College of Nursing, Yonsei University, 50-1 Yonsei-ro, Seodaemun-gu, Seoul, South Korea 03722 South Korea

**Keywords:** Health care, Korea, Leadership, Nursing organizational culture

## Abstract

**Background:**

Nurses’ promotive voice behavior, or the act of proactively suggesting improvements in the workplace, is crucial for enhancing healthcare services. Despite its importance, the factors that influence promotive voice, particularly within hierarchical environments, remain insufficiently explored. Understanding these factors is essential to creating work environments that encourage open communication and innovation among nursing staff. This study aimed to investigate the individual-level and unit-level factors that influence promotive voice among nurses, with a particular focus on the roles of age, unit tenure, power distance orientation, leadership style, and work climate.

**Methods:**

This study, employing a cross-sectional and correlational design, analyzed data from 1,255 registered nurses with over six months of clinical experience, all of whom were involved in direct patient care across 145 nursing units. Multilevel analysis was employed to examine the relationships between individual-level characteristics (age, unit tenure, power distance orientation) and unit-level factors (leadership style, work climate) on promotive voice behavior. The analysis accounted for the hierarchical structure of the data, with nurses nested within nursing units.

**Results:**

The analysis revealed that individual nurse’s age (B = 0.04, 95% CI [0.03, 0.05]) and unit tenure (B = 0.01, 95% CI [0.01, 0.02]) were positively associated with promotive voice, while power distance orientation was negatively associated with promotive voice (B = -0.08, 95% CI [-0.15, -0.01]). At the unit level, inclusive leadership style (B = 0.23, 95% CI [0.08, 0.38]) and a positive speaking-up work climate (B = 0.34, 95% CI [0.11, 0.57]) were significantly related to higher levels of promotive voice.

**Conclusions:**

This study highlights the significant influence of both individual characteristics and unit-level factors on promotive voice among nurses. The findings suggest that fostering inclusive leadership and a supportive speaking-up climate, while addressing hierarchical barriers, can enhance the propensity of nurses to engage in promotive voice. This, in turn, has the potential to improve patient outcomes and enhance overall organizational performance by promoting a culture of open communication and continuous improvement.

**Trial registration:**

Not applicable.

## Background

Nurses, who constitute the majority of hospital staff, play a pivotal role in enhancing healthcare services [1]. One important way they do this is through voice behavior, defined as the discretionary communication of ideas, suggestions, concerns, or opinions intended to improve organizational or unit functioning [2]. The significance of voice behavior has been increasingly recognized in healthcare literature and other industries due to its beneficial effects on organizational outcomes, including harm prevention, operational process improvement, and team learning [3, 4].

The organizational literature distinguishes between various types of voice. For instance, Liang et al. [5] introduced the concepts of promotive and prohibitive voice, and recent research in the nursing field has applied this distinction to healthcare settings [6]. Promotive voice is used by employees to proactively suggest improvements to enhance organizational or team functioning; whereas employees use prohibitive voice to raise concerns about behaviors or incidents that may negatively impact the organization [7]. Although both types of voice aim to benefit the organization [8], they differ in content and motivational drives [9]. Promotive voice is future-oriented, aiming to realize ideals and enact change, whereas prohibitive voice is more focused on identifying existing threats or problems [5].

Both types of voice are susceptible to suppression in healthcare settings due to hierarchical structures and fear of reprisal [2] but, they are triggered by distinct psychological mechanisms [5, 9, 10]. Promotive voice stems from a proactive mindset, oriented toward innovation and improvement, while prohibitive voice is often driven by risk aversion and a desire to prevent harm. This distinction has practical relevance in nursing practice, where promotive voice allows frontline staff to offer constructive suggestions to enhance care quality, patient safety, and organizational learning. Nevertheless, such contributions are often stifled by contextual and structural barriers, especially in high power distance environments that discourage upward communication [11]. Understanding the specific antecedents that foster promotive voice among nurses is essential for creating a work environment that supports innovation and continuous improvement. Despite growing recognition of the conceptual differences between promotive and prohibitive voice [11, 12], research into the unique antecedents of promotive voice remains limited [9].

Although voice behavior involves interpersonal communication, the workplace factors shaping it remain underexplored [13]. Most existing studies have focused primarily on individual-level predictors of voice behavior [14, 15]; for example, employees who feel respected tend to be proactively motivated to speak up [15]. Contextual factors such as leadership openness and a supportive voice climate also play a significant role in encouraging promotive voice, as trust and psychological safety within the work environment foster employees’ willingness to offer constructive suggestion [2, 16]. However, relatively few studies have examined how individual and contextual factors jointly influence promotive voice, suggesting a need for more research in clinical settings.

Among individual-level characteristics, nurses’ age, job longevity, and power distance orientation play a critical role in shaping their willingness to speak up. A recent review study highlighted that hierarchies in healthcare organizations restrict both the frequency of employee voice and the range of acceptable expressions [17]. This is particularly evident in East Asian cultures, where seniority-based hierarchies, largely determined by age and job tenure [18], influence health workers’ willingness to speak up [19]. In such cultural contexts, older employees are generally more respected and their opinions are highly valued, making it difficult for younger staff to express their concerns or opinions [20]. Moreover, previous research has shown that employees with longer work experience tend to engage more in voice behavior [21]. They tend to report greater voice efficacy and perceive lower social risk when speaking up, which further promotes their voice behavior [2]. Based on previous evidence, it is reasonable to hypothesize that age and unit tenure may affect nurses’ promotive voice.

### H1

Individual nurses’ age is positively related to promotive voice.

### H2

Individual nurses’ unit tenure is positively related to promotive voice.

Power distance orientation—the extent to which individuals accept unequal power distributions [11]—may influence employees’ promotive voice independently of demographic factors such as age and unit tenure. Power distance orientation is highly relevant to voice research because it provides insight into individuals’ reactions to the opinions of others [11]. Individuals with high power distance orientation tend to be more tolerant of social unfairness and unequal power distribution [22], as they are likely to hold superiors in high regard due to perceived status and superiority [23]. Such employees are more apt to accept and conform to the preexisting status quo, deferring to their leaders’ authority [24]. By contrast, individuals with low power distance orientation tend to express their opinions freely because they value individual rights and equality [22]. Drawing on the above evidence, we propose the following hypothesis:

### H3

Individual nurses’ power distance orientation is negatively related to promotive voice.

Employees’ perceptions of leadership style and work climate are recognized as important contextual factors influencing promotive voice. Direct supervisors/leaders can stimulate their staff to engage in voice behavior by actively inviting and appreciating staff input and building trusting relationships [25]. Since leaders set the level of interpersonal risk associated with speaking up and establish the relatedness necessary to motivate their expression of concerns [9], inclusive leadership style may be the main relational factor in organizations that encourage promotive voice. Inclusive leaders are characterized by their accessibility, openness, and acceptability for each team member’s point of view [26]. They value diverse perspectives by recognizing unique contributions and fostering a culture of open communication [27]. Compared to other leadership styles such as transformational, empowering, authentic, and servant leadership, inclusive leadership prioritizes creating a sense of belonging for employees and valuing their unique contributions [28, 29]. Inclusive leaders promote a work environment where every opinion is genuinely valued and facilitate proactive, innovative behavior among their staff [29]. Indeed, previous studies showed positive links between inclusive leadership and employee voice behavior in healthcare [18] and other industries [27]. Therefore, we put forward the following hypothesis:

### H4

Unit-level inclusive leadership is positively related to promotive voice.

Speaking-up climate captures the shared perceptions among employees that expressing constructive suggestions or concerns is encouraged, rewarded, and expected within the work environment [30]. While prior studies have primarily examined speaking-up climate in relation to patient safety concerns within healthcare settings [4, 31, 32], organizational research has emphasized its broader role in fostering promotive voice [2, 5, 16]. However, the specific impact of speaking-up climate on individual nurses’ promotive voice behavior remains underexplored. Our study addresses this gap by providing empirical evidence on the effect of speaking-up climate on nurses’ promotive voice, thereby contributing to a more nuanced understanding of the factors that encourage nurses to voice their insights and suggestions for better organizational functioning.

### H5

Unit-level speaking-up climate is positively related to promotive voice.

**Fig. 1:**
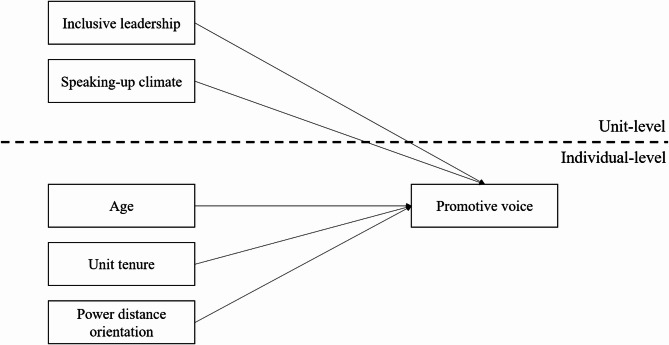
Hypothesized model

Figure 1 illustrates the hypothesized relationships described above. It distinguishes the effects of individual nurse characteristics, including age, unit tenure, and power distance orientation, from unit characteristics, such as inclusive leadership and speaking-up climate on nurses’ promotive voice.

## Methods

### Study design, sample, setting

This study was part of a larger research project that explored factors influencing patient and workplace safety in healthcare settings. This multilevel, correlational study used cross-sectional questionnaire data from 1,255 registered nurses employed in Korean hospitals, specifically those providing direct patient care in 145 units. Nurses eligible for the study were those with over six months of clinical experience in general (medical, surgical, or combined) and specialty units (critical care, perioperative, and emergency departments). The study excluded nurses holding managerial positions or working on a temporary basis. Further details on sampling methods are available elsewhere [6].

### Measures

#### Outcome variable

Promotive voice was measured using the five-item scale developed by Liang et al. [5], which has been used among Korean nurses, showing good reliability [33]. Participants responded on a five-point Likert scale ranging from 1 (*strongly disagree*) to 5 (*strongly agree*), with higher scores reflecting a higher level of promotive voice. In prior research, Cronbach’s alpha for the scale was 0.92 [33], and in the current study, the alpha was 0.93.

#### Individual-level nurse characteristics

For nurses’ individual characteristics, age and unit tenure were measured. Additionally, individual power distance orientation was assessed using the five-item instrument developed by Yoo et al. [34], which has showed good psychometric properties [35]. Responses were scored on a five-point Likert scale, ranging from 1 (*strongly disagree*) to 5 (*strongly agree*), with higher scores indicating stronger power distance orientation. The scale showed a Cronbach’s alpha of 0.79 in previous research [45] and 0.73 in this study.

#### Unit-level characteristics

Two unit-level characteristics were derived from nurse surveys by aggregating nurse-specific reports to describe unit characteristics [36]. To evaluate unit-level inclusive leadership, the nine-item scale developed by Carmeli et al. [26] was utilized. This scale has shown decent good reliability and validity in research involving Korean nurses [18]. Responses were scored on a five-point Likert scale ranging from1 (*strongly disagree*) to 5 (*strongly agree*), with higher scores reflecting greater levels of inclusive leadership by unit managers. Cronbach’s alpha of the scale was 0.95 in both prior research [18] and the current study.

Speaking-up climate was assessed using the five-item Speaking Up Climate for Safety, developed by Martinez et al. [37]. This scale was used to assess shared perceptions of how much the clinical environment supports speaking up about patient safety. This tool has been validated and shown reliable in studies with Korean nurses [38]. Responses were recorded on a five-point Likert scale, ranging from 1 (*strongly disagree*) to 5 (*strongly agree*), with higher scores indicating a more favorable speaking-up unit climate within the unit. The Cronbach’s alpha for this tool was reported as 0.78 in prior research [38] and 0.77 in the present study.

#### Control variables

We controlled for hospital location (metropolitan vs. non-metropolitan areas) and hospital size (number of beds) at Level 2.

#### Demographic information

Following demographic information was collected: gender, education level, years of nursing experience, and employment status.

### Data analysis

First, the data were evaluated to determine their suitability for the intended analytical methods. Participants’ characteristics and key study variables were summarized using descriptive statistics. To assess the relationships between study variables, Pearson correlation analysis were employed. We also checked for multicollinearity using the variance inflation factor, which yielded a value of 1.32, indicating no significant multicollinearity issues as per Hair et al. [39]. Given the data’s hierarchical nature, with registered nurses at Level 1 and nursing units at Level 2, a multilevel linear regression approach was adopted. All statistical analyses were conducted using STATA version 16.1, with a *p*-value of less than 0.05 indicating statistical significance.

## Results

### Sample characteristics

As shown in Tables 1, 1,255 nurses across 145 units participated in this study, averaging 8.7 nurses per unit. Most participants (*n* = 1,180, 94.0%) were women. The nurses had an average age of 31.2 years and had a mean of 7.5 years in nursing experience. A significant portion of the nurses (*n* = 1,169, 93.1%) held a baccalaureate or higher degree, and nearly all (*n* = 1,251, 99.7%) were employed in full-time positions. Over half of the nurses (*n* = 730, 58.2%) worked in general nursing units, such as medical, surgical or combined medical-surgical units. Regarding the hospitals involved, about two-thirds (*n* = 22, 64.7%) were situated in metropolitan areas of Korea.

**Table 1:** Sample characteristics of hospitals and nurses (*N* = 1,255)

Characteristic	Category	*n* (%) or M (SD)
Age (years)		31.2 (6.3)
Nursing experience (years)		7.5 (6.3)
Hospital tenure (years)		6.7 (6.0)
Unit tenure (years)		4.6 (4.3)
Gender	Women	1,180 (94.0)
	Men	75 (6.0)
Employment status	Permanent	1,251 (99.7)
	Temporary	4 (0.3)
Educational level	Diploma	86 (6.9)
	Baccalaureate or higher	1,169 (93.1)
Work unit	Medical, surgical, medical-surgical	730 (58.2)
	Specialty	525 (41.8)

Note. M, mean; SD, standard deviation; Specialty units included critical care, emergency, and perioperative units

### Preliminary analyses

The descriptive statistics and inter-variable correlations for the study’s key measures are presented in Table 2. The individual-level nurse characteristics, age and unit tenure, were both significantly correlated with promotive voice (*r* = 0.334, *p* < 0.001; *r* = 0.239, *p* < 0.01). Power distance orientation showed significantly negative correlations with inclusive leadership (*r* = -0.259, *p* < 0.001), speaking-up climate (*r* = -0.203, *p* < 0.001), and promotive voice (*r* = -0.068, *p* = 0.015). The unit-level characteristics, inclusive leadership and speaking-up climate, were significantly correlated with each other (*r* = 0.535, *p* < 0.001). Inclusive leadership was also positively associated with promotive voice (*r* = 0.287, *p* < 0.001).

**Table 2:** Summary of descriptive statistics and correlations coefficients for study variables (*N =* 1,255)

Variable	1	2	3	4	5		6
1. Age	—						
2. Unit tenure in years	0.491***	—					
3. Power distance orientation	0.003	–0.034	—				
4. Inclusive leadership	0.090**	0.051	–0.259***	—			
5. Speaking-up climate	0.066*	0.089**	–0.203***	0.535***	—		
6. Promotive voice	0.334***	0.239**	–0.068*	0.287***	0.299***		—
Mean	31.18	4.60	2.16	3.66	3.57	2.79	
Standard deviation	6.31	4.27	0.56	0.74	0.56	0.82	

***p < 0.001, **p < 0.01, *p < 0.05

### Multilevel analysis

A two-level model was utilized to investigate the impacts of individual nurse and unit characteristics on nurses’ promotive voice. The appropriateness of a multilevel model was evaluated against single-level linear regression models using the log likelihood ratio test. The test’s result suggests that there is marginal evidence that the two-level model provides a better fit than a linear model (χ2(1) = 2.70, *p* = 0.05). Considering the small yet present intraclass correlation of 0.02 and the risk of underestimating standard errors and increasing Type I error rates by neglecting the hierarchical nature of the data, a multilevel approach was determined to be appropriate [40].

The results of the multilevel linear regressions, as shown in Table 3, revealed that both individual characteristics and unit-level factors play significant roles in determining nurses’ promotive voice. Specifically, individual nurse’s age (B = 0.037, *p* < 0.001) and unit tenure (B = 0.002, *p* < 0.001) were positively associated with promotive voice, while power distance orientation (B = -0.076, *p* = 0.040) had a negative association with promotive voice. Additionally, both unit-level inclusive leadership (B = 0.229, *p* = 0.003) and speaking-up climate (B = 0.338, *p* = 0.004) exhibited positive relationships with promotive voice.

**Table 3:** Multilevel linear regression on promotive voice

	Coefficient	Standard error	*p*
Individual-level characteristics			
Age	0.037	0.004	< 0.001
Unit tenure	0.002	< 0.001	< 0.001
Power distance orientation	-0.076	0.038	0.040
Unit-level characteristics			
Inclusive leadership	0.229	0.079	0.003
Speaking-up climate	0.338	0.112	0.004

## Discussion

### Interpretation of findings

Previous studies on promotive voice have predominantly focused on individual-level antecedents, often neglecting the broader context of unit-level factors. An individual- vs. unit-level focus limits our understanding of promotive voice, particularly regarding the comparative influence of factors at these different levels [13]. To address this gap, our study examined both individual- and unit-level determinants of promotive voice among nurses. We discovered that both individual characteristics, such as age, unit tenure, and power distance orientation, and unit-level factors, including inclusive leadership and a speaking-up climate, significantly contribute to promotive voice among nurses.

Building on previous research [20, 21], our study provides additional evidence that both age and unit tenure are significant factors influencing nurses’ promotive voice. Our findings provide a more nuanced understanding of how hierarchical differences related to age and job tenure impact promotive voice by considering these variables within a single analytical model. Specifically, we found that nurses with older age and longer unit tenure are more likely to engage in promotive voice. This suggests that hierarchical norms in healthcare settings may empower senior staff to feel more entitled or confident in expressing suggestions for improvement. However, age and tenure are often closely correlated in such contexts, making it difficult to fully disentangle their individual effects. Future research should examine whether these relationships hold in other cultural settings, particularly those with differing norms regarding organizational hierarchy.

Another notable finding from our study is that individual-level power distance orientation negatively affects nurses’ promotive voice. The negative association suggests that nurses who accept hierarchical power differences are less likely to engage in promotive voice, possibly due to concerns about potential repercussions or a belief in the appropriateness of maintaining the status quo. This finding aligns with earlier research showing that high power distance hinders open communication [41]. This finding can be interpreted through the socially desirable responding theory [42], which explains that individuals with high power distance orientation tend to defer to superiors [12]. In contrast, employees with low power distance orientation value equality and are more inclined to contribute their suggestions for organizational improvements and innovation [11].

The significant positive relationships between inclusive leadership and speaking-up climate with promotive voice highlight the critical role of leadership and organizational context in encouraging nurses’ promotive voice. A supportive speaking-up climate can lower the psychological barriers associated with hierarchy, thereby enabling nurses to express constructive suggestions more freely. When staff collectively perceive that speaking up is encouraged and valued, they are more likely to believe their input will be taken seriously, which in turn fosters promotive voice behavior. Inclusive leadership further reinforces this climate by modeling openness, accessibility, and appreciation of diverse viewpoints [25–27]. Drawing on the broaden-and-build theory [43], consistent positive feedback and role modelling by inclusive leaders foster a work climate of positivity and proactive attitudes [44]. Positive interactions with peers and leaders broaden professional capacities and enable future-oriented thinking [44].

### Implications for practice

Our findings offer several actionable insights for healthcare leaders seeking to promote promotive voice among nurses. First, cultivating inclusive leadership is essential. Leaders who demonstrate openness, accessibility, and appreciation for diverse perspectives can encourage staff across different levels of seniority and tenure to contribute ideas and suggestions [45]. Second, organizations should create structured opportunities for voice, such as regular debriefings, suggestion systems, and formal feedback mechanisms that signal leadership’s commitment to staff input [44]. Finally, fostering a psychologically safe work environment—where speaking up is not only accepted but expected—can further encourage promotive voice. When employees feel safe to express their opinions without fear of negative consequences, they are more likely to speak up and contribute innovative ideas for improvement.

### Limitations

This study has a number of limitations. First, its cross-sectional design restricts the ability to infer causality. Second, the findings of this study may not be generalizable due to the regional focus. Future research should examine how these dynamics unfold in various cultural contexts or healthcare systems, as cultural norms and organizational structures may vary significantly across settings. Third, this study relied on self-report measures to assess promotive voice, which could introduce bias such as social desirability bias. Future studies could address this limitation by incorporating objective performance ratings or supervisor assessments. Additionally, investigating other individual characteristics, such as personality traits or communication skills, along with additional unit-level factors such as organizational policies, specific leadership styles, and team dynamics, could provide a deeper understanding of the factors that influence nurses’ promotive voice in healthcare settings.

## Conclusions

This study enhances our understanding of factors that influence promotive voice among nurses, highlighting the significance of both individual characteristics, such as age, tenure and power distance orientation; and unit factors, including inclusive leadership and a supportive speaking-up climate. The findings emphasize the critical role of fostering speak-up work climates where nurses are empowered to share their ideas and to contribute to innovation. To achieve this, healthcare organizations should prioritize leadership development programs that promote inclusivity and establish effective channels for open communication among staff. Addressing barriers such as hierarchical dynamics and power distance will ultimately lead to improved patient outcomes and enhanced organizational performance.

## Data Availability

The datasets generated and/or analysed during the current study are not publicly available due to them containing information that could compromise research participant consent but are available from the corresponding author on reasonable request.
